# Neural Signatures of Engagement and Event Segmentation during Story Listening in Background Noise

**DOI:** 10.1523/ENEURO.0385-25.2025

**Published:** 2026-01-09

**Authors:** Björn Herrmann, Aysha Motala, Ryan A. Panela, Ingrid S. Johnsrude

**Affiliations:** ^1^Rotman Research Institute, Baycrest Academy for Research and Education, North York, Ontario M6A 2E1, Canada; ^2^Department of Psychology, University of Toronto, Toronto, Ontario M5S 1A1, Canada; ^3^Department of Psychology, University of Western Ontario, London, Ontario N6A 5B7, Canada; ^4^Department of Psychology, University of Stirling, Stirling FK9 4LA, United Kingdom; ^5^School of Communication Sciences & Disorders, University of Western Ontario, London, Ontario N6G 1H1, Canada

**Keywords:** event segmentation, functional magnetic resonance imaging, intersubject correlation, listening effort, naturalistic speech processing, speech masking

## Abstract

Speech in everyday life is often masked by background noise, making comprehension effortful. Characterizing brain activity patterns when individuals listen to masked speech can help clarify the mechanisms underlying such effort. In the current study, we used functional magnetic resonance imaging (fMRI) in humans of either sex to investigate how neural signatures of story listening change in the presence of masking noise. We show that, as speech masking increases, spatial and temporal activation patterns in auditory regions become more idiosyncratic to each listener. In contrast, spatial activity patterns in brain networks linked to effort (e.g., cingulo-opercular network) are more similar across listeners when speech is highly masked and less intelligible, suggesting shared neural processes. Moreover, at times during stories when one meaningful event ended and another began, neural activation increased in frontal, parietal, and medial cortices. This event-boundary response appeared little affected by background noise, suggesting that listeners process meaningful units and, in turn, the gist of naturalistic, continuous speech even when it is masked somewhat by background noise. The current data may indicate that people stay engaged and cognitive processes associated with naturalistic speech processing remain intact under moderate levels of noise, whereas auditory processing becomes more idiosyncratic to each listener.

## Significance Statement

Everyday listening often happens in noisy environments. Using brain imaging, we examined how people's brain activity changes when listening to naturalistic stories in background chatter. We found that as the noise increased, brain activity in auditory areas became more unique to each person, while regions (anterior insula and cingulate) linked to effort showed more similar patterns across listeners. A large number of brain regions in frontoparietal and attention networks responded strongly when one part of the story ended and another began, and this effect remained stable even with moderate noise. These findings reveal distinct neural systems involved in naturalistic speech listening and suggest that people continue to make sense of stories, even when words are partly masked by background sounds.

## Introduction

Speech in everyday life is often degraded or masked by background noise, which makes comprehension cognitively demanding and effortful (Eckert et al., 2016; Pichora-Fuller et al., 2016; Peelle, 2018; Herrmann and Johnsrude, 2020a). Listening effort is considered an early sign of age-related hearing loss (Pichora-Fuller et al., 2016; Helfer and Jesse, 2021) and characterizing what happens in the brain when individuals listen to degraded or masked speech may clarify the mechanisms underlying listening effort (Eckert et al., 2016; Johnsrude and Rodd, 2016). Research thus far has focused mainly on the neural processes related to speech degradation/masking while individuals listen to short, disconnected sentences (Obleser and Kotz, 2010; Okada et al., 2010; Wild et al., 2012b; Ritz et al., 2022). Such speech materials resemble little the more continuous, story-like spoken speech individuals often encounter (Jefferson, 1978; Mullen and Yi, 1995; Bohanek et al., 2009). Continuous speech requires a listener to mentally organize the speech stream into meaningful units that span across sentences and enables shared immersive experiences such as suspense, anticipation, empathy, and enjoyment (Speer et al., 2004; Whitney et al., 2009; Michelmann et al., 2021). The lack of a coherent narrative in disconnected sentences limits our understanding of the neurocognitive processes underlying continuous speech comprehension, engagement, and the segmentation of meaningful information across extended timescales.

Research using functional magnetic resonance imaging (fMRI) to study the responses to sentences masked with noise has shown activations in the cingulo-opercular network (e.g., cingulate cortex, insula), prefrontal cortex, and parietal networks as degradation/masking of speech increases and listening becomes more effortful, whereas activity in the anterior and posterior temporal cortex increases as speech becomes more intelligible (Davis and Johnsrude, 2003; Scott et al., 2006; Obleser and Kotz, 2010; Wild et al., 2012b; Scott and McGettigan, 2013; Evans et al., 2016; Ritz et al., 2022). Whether the activation patterns observed for degraded/masked sentences also hold for more naturalistic, continuous speech listening is unclear. The current study explores how the neural systems involved in effort operate under more ecologically valid listening conditions.

Other fMRI work has focused on neural processes during the perception of continuous, naturalistic stimuli, such as movies and spoken speech in quiet (Nummenmaa et al., 2014; Chen et al., 2017; Regev et al., 2019; Hamilton and Huth, 2020). When materials are engaging, neural activity in wide networks, including the default mode and frontoparietal networks, synchronizes across observers or listeners (Honey et al., 2012; Nummenmaa et al., 2014; Chen et al., 2017; Nguyen et al., 2019; Regev et al., 2019). This neural synchronization, indexed as intersubject correlation (ISC) of neural activity (Hasson et al., 2010; Nastase et al., 2019), can be quantified temporally as the similarity in the time course of neural activity across listeners and spatially as the similarity in the spatial distribution of brain activity across listeners. Both measures complementarily capture shared neural processing during naturalistic stimulation and increase as immersive engagement with and shared understanding of the materials increase (Schmälzle et al., 2015; Nguyen et al., 2019; Song et al., 2021). Behavioral data and scalp-recorded electroencephalography (EEG) further suggest that engagement with spoken stories may be unaffected by moderate background noise (Herrmann and Johnsrude, 2020b; Irsik et al., 2022b; Yasmin et al., 2023). However, scalp EEG is ill suited to disentangle activity from different neural systems that may differ in the nature and magnitude of shared activity and how these change when speech comprehension is effortful due to background noise. The present fMRI study provides a means to distinguish distinct neural networks. We aim to identify regions in which activity becomes more idiosyncratic (less synchronized) across listeners as speech masking increases, reflecting individual responses to increasing listening challenges. We also aim to identify regions that show increased synchronization when speech is masked, reflecting a common response.

In the broader literature, engagement and effort may be related constructs. Engagement often refers to a state of absorbed, enjoyable, and sustained attention during meaningful stimulation, commonly indexed behaviorally as subjective ratings (Busselle and Bilandzic, 2009; Kuijpers et al., 2014; Herrmann and Johnsrude, 2020b; Mathiesen et al., 2024) or neurally as intersubject correlation (ISC) of activity in widespread networks (Hasson et al., 2010; Schmälzle et al., 2015; Nastase et al., 2019; Nguyen et al., 2019; Song et al., 2021; Irsik et al., 2022b). In contrast, effort involves the investment of cognitive resources when processing becomes challenging, often associated with activity in the cingulo-opercular and frontoparietal networks (Eckert et al., 2016; Johnsrude and Rodd, 2016; Peelle, 2018). Both constructs rely on attention, but they differ in subjective experience. Engagement is often pleasant and absorbing, whereas effort is associated with strain and cognitive load. Our recent model of listening engagement suggests that both share overlapping processes, with differences emerging primarily in the magnitude of resource investment and the accompanying experience (enjoyment vs demand; Herrmann and Johnsrude, 2020a). The present study provides the first opportunity to examine how neural systems and intersubject synchronization behave across conditions that vary in listening difficulty, allowing us to map activity of regions previously associated effort and engagement during naturalistic speech perception.

Although natural environments unfold continuously, individuals mentally organize them into discrete, temporally extended events that reflect the gist of information over several tens of seconds to minutes (Zacks et al., 2007; Zacks and Swallow, 2007). Individuals tend to agree on when one event ends and another one begins, henceforth referred to as an event boundary (Kurby and Zacks, 2008; Richmond et al., 2017; Sasmita and Swallow, 2022; Michelmann et al., 2025). This across-individual agreement is consistent with the shared neural activity patterns across individuals observed through ISC (Hasson et al., 2004, 2010; Nastase et al., 2019). The accurate mental organization of natural environments into meaningful events is associated with better recall of relevant information at a later time (Zacks et al., 2006; Sargent et al., 2013; Kurby and Zacks, 2018; Newberry and Bailey, 2019). Critically, neural activity transiently increases around the time of an event boundary, potentially reflecting the increased processing demands associated with updating mental representations at an event boundary (Speer et al., 2007; Whitney et al., 2009; Zacks et al., 2010; Ben-Yakov and Henson, 2018; Barnett et al., 2024). How neural activity associated with event-boundary processing changes when individuals listen to spoken stories under varying degrees of background masking noise is unknown, but if it reflects overarching story comprehension, then it should be somewhat robust to speech masking (Yasmin et al., 2023). The current study is the first to examine how neural signatures of event segmentation during listening are affected by background noise.

In the current fMRI study, we analyze blood oxygenation level-dependent (BOLD) signal to investigate how neural signatures of engagement and event segmentation change while individuals listen to naturalistic, spoken stories masked by different degrees of background babble that degrades intelligibility but may not affect comprehension as much. Analyses focus on masking-related changes in overall activation, intersubject synchronization of spatial and temporal patterns of brain activity, and neural responses at the times of event boundaries. Together, the current study provides insights into how neural systems associated with effort, engagement, and event segmentation operate under ecologically valid listening conditions, thereby extending our understanding of the neural processes involved in speech comprehension in continuous, noisy environments.

## Materials and Methods

### Participants

Forty adults participated in the current study (median age, 23 years; age range, 17–34 years; 16 male, 24 female). Data from five additional individuals were recorded but excluded from data analysis because a few volumes during functional imaging were not recorded (*n* = 1) or behavioral performance was at chance level, suggesting inattentive listening to the spoken stories (*n* = 4). Participants were native English speakers or learned English before the age of 5 years. Participants reported having no hearing impairment. Participants gave written informed consent prior to the experiment and were paid $20 CAD per half-hour for their participation. The study was conducted in accordance with the Declaration of Helsinki, the Canadian Tri-Council Policy Statement on Ethical Conduct for Research Involving Humans (TCPS2-2014), and was approved by the Research Ethics Board of the University of Western Ontario.

### Acoustic stimuli and procedures

The experiment was run using Psychtoolbox (version 3.0.14) on a Lenovo ThinkPad W550s laptop under Windows 7. Visual stimuli were presented to participants in the MR scanner through a mirror system. Acoustic stimuli were presented via a Steinberg UR22 external sound card and played to participants through Sensimetrics MR compatible headphones (model S14). Auditory stimuli were presented at a comfortable listening level, determined at the beginning of the fMRI session by playing a ∼1 min story in the scanner.

Participants listened to three stories from the story-telling podcast “The Moth” (themoth.org). The selected stories were “Reach for the stars one small step at a time” by Richard Garriott (13:26 min, 2,586 words, male speaker), “The bounds of comedy” by Colm O’Regan (10:03 min, 1,777 words, male speaker), and “Nacho challenge” by Omar Qureshi (11:10 min, 1,833 words, male speaker). The Moth stories are about human experiences and life events, and they are intended to create an engaging and enjoyable listening experience. The Moth stories mirror speech in everyday life, such as disfluencies, filler-words, sentence fragments, corrections, unintentional pauses, and more flexible grammar (Tree, 1995; Bortfeld et al., 2001; Panela et al., 2024). The Moth stories are increasingly used in behavioral and neuroimaging studies due to their naturalness (Ki et al., 2016; Simony et al., 2016; Regev et al., 2019; Herrmann and Johnsrude, 2020b; Irsik et al., 2022a,b; Panela et al., 2024).

Twelve-talker babble from the Revised Speech in Noise test (Bilger, 1984; Bilger et al., 1984) was added to the stories at different speech-clarity conditions. Twelve-talker babble is a standardized masker in speech-in-noise tests that simulates a crowded restaurant, while not permitting the identification of individual words in the masker (Mattys et al., 2012). The amplitude of a twelve-talker babble is relatively stationary over time and thus provides continuous speech masking (Bilger, 1984; Bilger et al., 1984; Wilson, 2003; Wilson et al., 2012).

**Figure 1.**
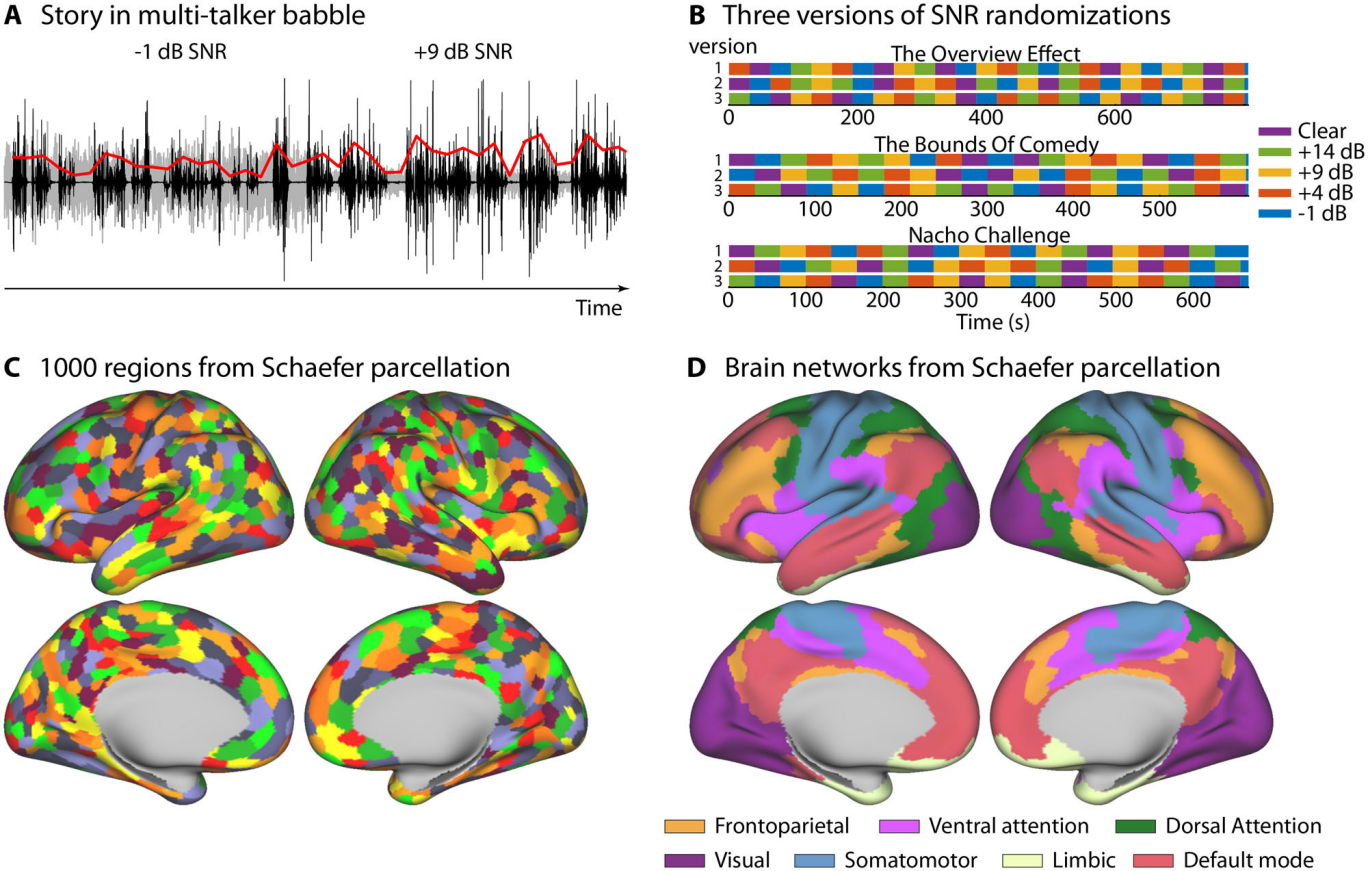
Illustration of the story-in-noise stimulation and brain parcellation. ***A***, A story was played continuously and the signal-to-noise ratio of the speech to the babble masker changed every 30–33 s. For visualization, the speech and babble are displayed separately (but were added for the experiment). Speech is displayed in black. The babble masker is displayed in gray. The red line reflects the speech envelope used for correlations with the BOLD signal (the envelope was linearly amplified for visualization). ***B***, Randomization of SNR conditions for the three stories and three randomization versions. ***C***, Brain parcellation of the Schaefer atlas into 1,000 regions. The color coding is arbitrary and meant to distinguish between different regions. Colors repeat for different regions. ***D***, Grouping into seven networks of the Schaefer atlas (Yeo et al., 2011; Schaefer et al., 2018).

In the current study, the speech-clarity level changed pseudorandomly every 30–33 s among five conditions: clear speech, +14 dB, +9 dB, +4 dB, −1 dB signal-to-noise ratio (SNR; Fig. 1), such that a particular speech-clarity condition could not repeat immediately. In a previous study using the same stories, speech intelligibility ranged from 95 to 55% correctly heard words for clear speech and speech at +12 dB, +7 dB, +2 dB, −3 dB SNR (Irsik et al., 2022b). To achieve somewhat similar intelligibility levels in the current study, we increased the SNR levels by 2 dB relative to the previous work because the MRI scanner generates minor additional background noise. SNR was manipulated by adjusting the sound level of both the story and the babble masker to ensure that the overall sound level remained constant throughout a story and across stories. Four 30 and 33 s segments per speech-clarity condition were presented for stories by Colm O’Regan and Omar Qureshi, respectively, and five 32 s segments per speech-clarity condition were presented for the story by Richard Garriott.

The current study aimed to investigate intersubject correlation of neural activity, which requires that each participant listens to the same story segment masked by the same speech-clarity level (Hasson et al., 2010; Nastase et al., 2019; Regev et al., 2019; Irsik et al., 2022b). The randomization of speech-clarity levels was thus fixed across participants for each story. To ensure that specific parts of a story were not confounded with a specific speech-clarity condition, three different versions of speech-clarity randomization were created for each story, and participants were assigned to one of the three versions. The order in which stories were presented was counterbalanced across participants. The three versions were pregenerated due to long generation times, and the same audio files were used for each participant of a specific group.

After each story, participants performed a brief story-comprehension task to assess whether participants had attentively listened. Eight statements were visually presented, and participants had to indicate via button press whether the statement was correct or incorrect. The proportion of correct responses was calculated and data from a participant were excluded if their proportion of correct responses was at or below chance level (0.5) for any of the stories. Data from four participants were excluded for this reason.

### Recording of MRI data

Magnetic resonance imaging (MRI) was conducted using a 3T Siemens MAGNETOM Prisma Fit scanner (Siemens) with a 32-channel head-coil at the Centre for Functional and Metabolic Mapping at the University of Western Ontario. Participants were comfortably positioned in the bore and wore air-conduction headphones (Sensimetrics S14).

Functional images, using multiband echoplanar imaging (Feinberg et al., 2010; Moeller et al., 2010; Barth et al., 2016) with an acceleration factor of 3, were acquired in 48 slices (interleaved), covering most of the brain, including temporal, frontal, parietal, and occipital cortices (subcortical regions and the cerebellum were not included systematically). The sequence was set up with an echo time (TE) of 30 ms, a flip angle of 40°, and a repetition time (TR) of 1.0 s. The matrix was 84 × 84 pixels (7/8 partial phase) with a field of view of 208 mm^2^. The in-plane resolution was 2.5 × 2.5 mm^2^. The slice thickness was 2.5 mm. Functional images were acquired in three independent runs which were separated by a short break of ∼30–60 s in which scanning was discontinued. The numbers of volumes acquired were 608, 676, and 811 for the story by Colm O’Regan, Omar Qureshi, and Richard Garriott, respectively. The number of volumes per story included ∼5 s (5 volumes) after the story ended to account for the slow temporal evolution of the hemodynamic response function during data analysis (Buckner, 1998; Lindquist et al., 2009; Taylor et al., 2018).

For each participant, a T_1_-weighted anatomical image of the brain was recorded for coregistration during data preprocessing. The T_1_-weighted image was acquired using the following parameters: TR, 2.3 s; TE, 2.98 ms; TI, 0.9 s; number of slices, 176; matrix, 256 × 256; field of view, 256 mm^2^; and a voxel size of 1 mm × 1 mm × 1 mm.

### Preprocessing of data

Data were preprocessed using SPM12 (Friston, 2007; Wellcome Trust Centre for Neuroimaging) and custom MATLAB scripts. For each of the three functional runs per participant, the preprocessing comprised slice-time correction (accounting for multiband slice acquisition), rigid-body spatial realignment and unwarping, segmentation of the T_1_-weighted image, coregistration to the T_1_-weighted image according to spatial normalization parameters from segmentation, normalization to the Montreal Neurological Institute (MNI) space (MNI152NLin2009cAsym; Fonov et al., 2009), and interpolation to a 2 mm × 2 mm × 2 mm voxel size. Spatial smoothing was not applied because most analyses focused on the averaged BOLD signal within anatomically defined brain regions (essentially smoothing across voxels). For those analyses that focused on the voxel-wise BOLD signal, we were interested in the spatial activation patterns across voxels within a brain region and thus wanted to avoid reducing potentially relevant variability. Motion-related artifacts were addressed by regressing out the six standard motion parameter time series, which include translation (*x*, *y*, *z*) and rotation (roll, pitch, yaw), using the 3dDetrend function from AFNI (Analysis of Functional NeuroImages) software (Cox, 1996; Cox and Hyde, 1997). Preprocessing resulted in three 4D functional datasets, one for each story, that were used for subsequent data analysis.

### Data reduction to regions of whole-brain parcellation

To reduce data dimensionality and computational times, all data analyses focused on regions from a whole-brain parcellation. In the current study, the Schaefer atlas was used to obtain 500 brain regions per hemisphere (Schaefer et al., 2018), which are further grouped into seven networks (Fig. 1*B*; Yeo et al., 2011; Schaefer et al., 2018). The Schaefer atlas was chosen because the regions are of approximately similar size, making this atlas particularly useful for dimensionality reduction while covering the whole cortex.

For most analyses, BOLD activity time courses from different voxels within a region were averaged to obtain one time course per Schaefer region. The averaged time course for each region was the basis for subsequent analyses (temporal intersubject correction; event-boundary analysis). This enables spatial resolution at the level of brain regions, while also enabling computationally intense calculations, such as temporal intersubject correlation analysis. The only exception was the analysis of spatial intersubject correlation, for which the BOLD signal was averaged across time separately for each voxel and analyses focused on the mean BOLD signal for each voxel within a Schaefer region (see below).

The granularity of the 500 Schaefer brain regions per hemisphere is important for gaining insight into the spatial distribution of speech-clarity effects described below. In contrast, visualizing time course is better achieved at an aggregate across regions. Hence, to visualize BOLD signal time courses for some analyses (see event-boundary analyses below), response time courses were further averaged across individual Schaefer regions, separately for each of the seven networks (Fig. 1*B*; Yeo et al., 2011; Schaefer et al., 2018). Hence, the further reduction of data to the seven networks mainly served time course visualization purposes and explorative analyses. The details for each specific analysis are described in the next section.

### Data and statistical analysis

#### BOLD activity to the amplitude of the speech envelope

Our initial data analysis focused on BOLD responses in each region of the Schaefer atlas to the acoustic properties of the speech signal (Honey et al., 2012; Rowland et al., 2018). The amplitude envelope was obtained for each story by calculating the absolute value of the clear speech signal. To match the sampling frequency of the BOLD signal (1 s TR), the mean amplitude envelope within 1 s time bins was calculated for each story. Separately for each story, the resampled amplitude envelope was convolved with a canonical hemodynamic response function (Lindquist et al., 2009; Honey et al., 2012). A design matrix was created that contained a unique regressor for each story's convolved envelope and a unique intercept regressor for each story. One general linear model was calculated for each participant and region of the Schaefer atlas (Friston et al., 1994), using the BOLD activity time course (concatenated for the three stories) as the predicted variable and the design matrix as the predictor. A contrast vector was multiplied with the estimated coefficients, such that the story regressors were set to 1 and the other regressors to 0. This resulted in one contrast coefficient for each participant and region of the Schaefer atlas. Positive values indicate a larger BOLD signal with increasing speech amplitude, whereas negative values indicate a larger BOLD signal with decreasing speech amplitude. For each Schaefer region, contrast coefficients were tested against zero using a one-sample *t* test (group-level analysis) and the resulting *t* value was converted to a *z*-score. *z*-scores were mapped onto a partially inflated standard brain surface and visualized using the workbench environment of the Human Connectome Project (Marcus et al., 2011). *z*-scores were thresholded at 3.89, corresponding to a Bonferroni-corrected significance threshold (alpha value of 0.05 divided by 1,000 regions, converted to a *z*-score equals 3.89).

#### Effect of speech clarity on BOLD activity

Separately for each region of the Schaefer atlas, we analyzed the effect of speech masking on the BOLD activity to investigate whether story materials lead to similar neural activity changes compared with the activity changes reported previously for degraded/masked spoken sentences (Scott and Johnsrude, 2003; Scott et al., 2006; Wild et al., 2012a,b; Scott and McGettigan, 2013; Ritz et al., 2022). For this analysis, a design matrix with 18 regressors was created. The design matrix contained one unique regressor for each of the speech-clarity conditions (clear speech, +14 dB, +9 dB, +4 dB, −1 dB SNR) for each of the three stories and one intercept regressor for each story. The 15 speech-condition regressors were convolved with a canonical hemodynamic response function (Lindquist et al., 2009). One general linear model was calculated for each participant and region of the Schaefer atlas (Friston et al., 1994), using the BOLD activity time course (concatenated for the three stories) as the predicted variable and the design matrix as the predictor. We examined the linear relationship between BOLD activity and speech-clarity conditions by multiplying a contrast vector with the estimated coefficients (Pearson and Hartley, 1970; Poldrack et al., 2011). Values of the contrast vector were coded as −2, −1, 0, 1, 2 for the clear speech, +14 dB, +9 dB, +4 dB, and −1 dB SNR conditions, respectively, whereas the other regressors were set to 0. This resulted in one contrast coefficient for each participant and region. Positive values indicate an increase in BOLD activity with increasing speech masking and associated listening effort, whereas negative values indicate an increase in BOLD signal with decreasing speech masking and associated intelligibility. For each Schaefer region, contrast coefficients were tested against zero using a one-sample *t* test (group-level analysis) and the resulting *t* value was converted to a *z*-score, mapped onto a partially inflated standard brain surface, and threshold at 3.89 (Bonferroni-corrected significance threshold).

#### Intersubject correlation analysis

One important neural signature of the processing of naturalistic, continuous stimuli is the degree to which neural activity patterns are similar across participants, referred to as intersubject correlation (ISC; Hasson et al., 2004, 2010; Nastase et al., 2019; Regev et al., 2019). We focused on two types of ISC analyses, capitalizing on spatial and temporal pattern similarity (Nastase et al., 2019; Regev et al., 2019; Lee and Chen, 2022). Spatial ISC reveals the degree to which the neural activation patterns of different voxels within a brain region are shared among participants. Temporal ISC reveals the degree to which neural activation of a brain region evolves similarly over time across different individuals. ISC analyses were separately calculated for the three subgroups of participants—that is, people who listened to the same randomization of speech-clarity conditions—before conducting group analyses involving all participants. For both spatial and temporal ISC, the whole BOLD signal time courses were first time shifted by 5 s to account for the hemodynamic response delay (Buckner, 1998; Lindquist et al., 2009; Taylor et al., 2018).

For the spatial ISC analysis, the BOLD signal for each 30–33 s speech-clarity segment was averaged over time, separately for each voxel, and subsequently averaged across the segments with the same speech-clarity level in a story. The mean signal across the voxels within a Schaefer region was subtracted from the activity value of each voxel within that region (i.e., mean-centered), separately for each speech-clarity condition and story. The activity values for the three stories were subsequently concatenated, leading to one activity vector per Schaefer region, speech-clarity level, and participant. For each Schaefer region and speech-clarity level, a leave-one-out procedure was implemented to calculate an ISC value for each participant. The activity vector of one participant was left out, and the activity vectors for the other *n* − 1 participants were averaged. The activity vector of the participant who was left out was correlated with the averaged activity vector of the *n* − 1 participants using Spearman correlation, and the resulting correlation value was used as the ISC value for the participant who was left out. The procedure was repeated such that each participant was left out once. The leave-one-out procedure was calculated separately for each subgroup of participants. These calculations resulted in one ISC value for each participant, speech-clarity condition, and brain region of the Schaefer atlas.

For the temporal ISC analysis, BOLD signal time courses for each Schaefer region were separated into individual 30–33 s speech-clarity segments. The mean BOLD signal for a given 30–33 s segment was subtracted from the BOLD signal at each sample of the segment (i.e., mean-centered). The mean-centered BOLD signal time courses of individuals segments and the three stories were concatenated, separately for each of the five speech-clarity conditions. For each Schaefer region, a leave-one-out procedure was implemented to calculate an ISC value for each participant and speech-clarity condition. The concatenated time course of one participant was left out, and the time courses for the other *n* − 1 participants were averaged. The time course of the participant who was left out was correlated with the averaged time course of the *n* − 1 participants using Spearman correlation, and the resulting correlation value was used as the ISC value for the participant who was left out. The procedure was repeated such that each participant was left out once. The leave-one-out procedure was calculated separately for each subgroup of participants. These calculations resulted in one ISC value for each participant, speech-clarity condition, and Schaefer brain region.

To assess whether speech clarity affects spatial and temporal ISC, a quadratic function—capturing linear and quadratic trends—was fit to relate ISC values to speech-clarity conditions (coding −2, −1, 0, 1, 2 for the clear, +14 dB, +9 dB, +4 dB, and −1 dB SNR conditions, respectively). We focused here on both linear and quadratic trends because the quadratic effect can capture changes in ISC that are driven mainly by high or low speech clarity conditions, which the linear effect may not capture well. There are a few instances where this is the case (see below). Linear and quadratic coefficients from the quadratic function fit were estimated separately for each participant and brain region. A positive linear coefficient indicates higher ISC as speech masking increases (SNR decreases), whereas a negative linear coefficient indicates lower ISC values as speech masking increases. A positive quadratic coefficient indicates higher ISC for the two end points or one end point of the five speech-clarity conditions relative to the other conditions. The linear and quadratic coefficients were tested against zero using a one-sample *t* test, separately for each brain region. The resulting *t* value was converted to a *z*-score, mapped onto a partially inflated standard brain surface, and thresholded at 3.89 (Bonferroni-corrected significance threshold).

In some previous work using ISC, permutation analyses to calculate chance level ISC have been conducted (Honey et al., 2012). In the current study, the calculation of ISC *z*-scores for each participant and condition (instead of ISC values) by permuting spatial activation patterns and time-shifting temporal activation patterns, and subsequent statistical analyses for the ISC *z*-scores, led to qualitatively similar results as for the analyses described in the previous paragraphs. For simplicity and better interpretability of the results, we limit reporting of the results to analyses for ISC values and do not report ISC *z*-scores.

#### Activation to event boundaries

The times at which event boundaries occurred in a story were determined to investigate neural activation related to event boundaries. To this end, a transcription was obtained manually for each story. The transcription was used with OpenAI's large language model GPT-4 (Generative Pre-trained Transformer 4; OpenAI et al., 2023) to identify event boundaries for each story. Previous work has shown that segmentation of stories into distinct events using OpenAI's GPT closely aligns with event segmentation by humans (Michelmann et al., 2025; Panela et al., 2025). The segmentation approach was implemented in Python 3.11.5 (van Rossum and Drake, 2009) using OpenAI's Application Programming Interface (API). The following prompt was input to OpenAI's model to identify event boundaries (see also Michelmann et al., 2025; Panela et al., 2025): “An event is an ongoing coherent situation. The following story needs to be copied and segmented into large events. Copy the following story word-for-word and start a new line whenever one event ends and another begins. This is the story: …”. After this prompt, the full transcription of a story was inserted (without paragraph breaks or other formatting that could bias segmentation), followed by an additional prompt to refresh and reiterate the instructions: “This is a word-for-word copy of the same story that is segmented into large event units” (Michelmann et al., 2025; Panela et al., 2025). The temperature parameter of the model was set to 0 to facilitate a deterministic and reproducible output (Panela et al., 2025; max_tokens was set to 4,096). Through this procedure, 44 events and corresponding boundaries were identified across the transcriptions of the three stories (story transcripts and OpenAI-identified event boundaries; see https://osf.io/2z7vr).

To obtain the times at which event boundaries occurred in the auditory story, we used the online implementation of Clarin's forced alignment software. The forced alignment software provides the onset time for each word in a story using the story audio files and story transcriptions (https://clarin.phonetik.uni-muenchen.de/BASWebServices/; Kisler et al., 2017; incorrect estimations were manually corrected). The onset times of the words that OpenAI's GPT-4 had identified as event boundaries (i.e., the beginning of a new event) were used for time-locking the BOLD signals for analysis. The center or midpoint of an event was used as a nonboundary control condition. Times of the event centers were calculated as the midpoint between two event boundaries. We reasoned that if neural responses to event boundaries reflect mental segmentation into meaningful story units, the responses should be larger than activity to event centers (Whitney et al., 2009). See also Extended Data Figure 5-1 for results using nonboundary sentence-onset times as a control.

Previous work shows a transient response peaking ∼5 s following an event boundary (Speer et al., 2007; Zacks et al., 2010; Reagh et al., 2020), consistent with the current study. To capture the full response time course around event boundaries and the control condition, we identified for each Schaefer region the 20 s epochs in the BOLD time courses centered on the times of event boundaries and event centers. Epochs started 10 s before and lasted for 10 s after, and all epochs were averaged separately for event boundary and event center, ignoring speech clarity for this analysis. The magnitude of the event-boundary response has previously been quantified by contrasting post- to pre-event boundary activity (Whitney et al., 2009). Following this approach, the BOLD signal across the −7 to −1 s time window was averaged and served as a baseline (referred to as “pre” period) against which to contrast the averaged BOLD signal across the +1 to +7 s time window (“post” period). Contrasts were created for the average post-minus-pre response for event boundary and event center conditions for each participant and brain region of the Schaefer atlas. For each Schaefer region, a paired-samples *t* test was calculated, testing whether the post-minus-pre response difference differed between event boundaries and event centers. The resulting *t* values were converted to *z*-scores, mapped onto a partially inflated standard brain surface, and thresholded at 3.89 (Bonferroni-corrected significance threshold). Positive values indicate a larger response around event boundaries than event centers, whereas negative values indicate a smaller response.

To visualize time-locked response time courses for the different conditions (event boundary, event center), the full (−10 to +10 s) response time courses were averaged across regions, separately for the seven networks of the Schaefer atlas (Fig. 1*B*; Yeo et al., 2011; Schaefer et al., 2018). For each network, we also explored differences in the time courses between responses to event boundaries and event centers. For this explorative analysis, a paired-samples *t* test was calculated for each time point, contrasting event-boundary versus event-center conditions. For this explorative analysis, false discovery rate (FDR) was used to correct for multiple comparisons across time points (Benjamini and Hochberg, 1995; Genovese et al., 2002).

To investigate whether neural responses to event boundaries are affected by the level of speech masking, epochs were split into two groups depending on whether they occurred during high (clear, +14 dB SNR) or low speech-clarity segments (+4 dB, −1 dB SNR). Splitting speech-clarity conditions into two groups ensured that a sufficient number of event-boundary epochs per high (*N* = 17, 16, 14 for the three versions of speech-clarity randomizations, respectively) and low (*N* = 19, 21, 21) speech-clarity group were available for analysis. The BOLD signal across the −7 to −1 s time window was averaged and subtracted from the averaged BOLD signal across the +1 to +7 s time window, separately for high and low speech-clarity conditions. Then, the post-minus-pre activity difference for the low speech-clarity condition was subtracted from the post-minus-pre activity difference for the high speech-clarity condition, resulting in an interaction measure that reflects the change in event-boundary activation (post vs pre) with speech clarity. A positive value indicates a larger event-boundary response for high compared with low speech clarity. Grouping into low and high speech-clarity groups and similar calculation were also conducted for event centers (high: *N* = 19, 20, 21; low: *N* = 19, 16, 15; for the three versions of speech-clarity randomizations, respectively). For statistical analyses, we compared the speech-clarity-related change in event-boundary response with the speech-clarity-related changes in event-center responses using a paired-samples *t* test, separately for each region of the Schaefer atlas. This contrast reflects the interaction between time window (post, pre), speech clarity (high, low), and condition (event boundary, event center). *t* values were converted to *z*-scores, mapped onto a partially inflated standard brain surface, and thresholded at 3.89 (Bonferroni-corrected significance threshold).

For visualization, response time courses were averaged across the regions in each Yeo network, separately for the speech-clarity levels (high, low) and conditions (event boundary, event center). For explorative purposes, BOLD signal differences between high and low speech-clarity levels were also statistically examined for each network. A paired-samples *t* test was calculated for each time point, contrasting high versus low speech-clarity levels, separately for event boundary and event center conditions. For this explorative analysis, FDR was used to correct for multiple comparisons across time points (Benjamini and Hochberg, 1995; Genovese et al., 2002).

## Results

### BOLD signal changes with the changes in the speech envelope

**Figure 2.**
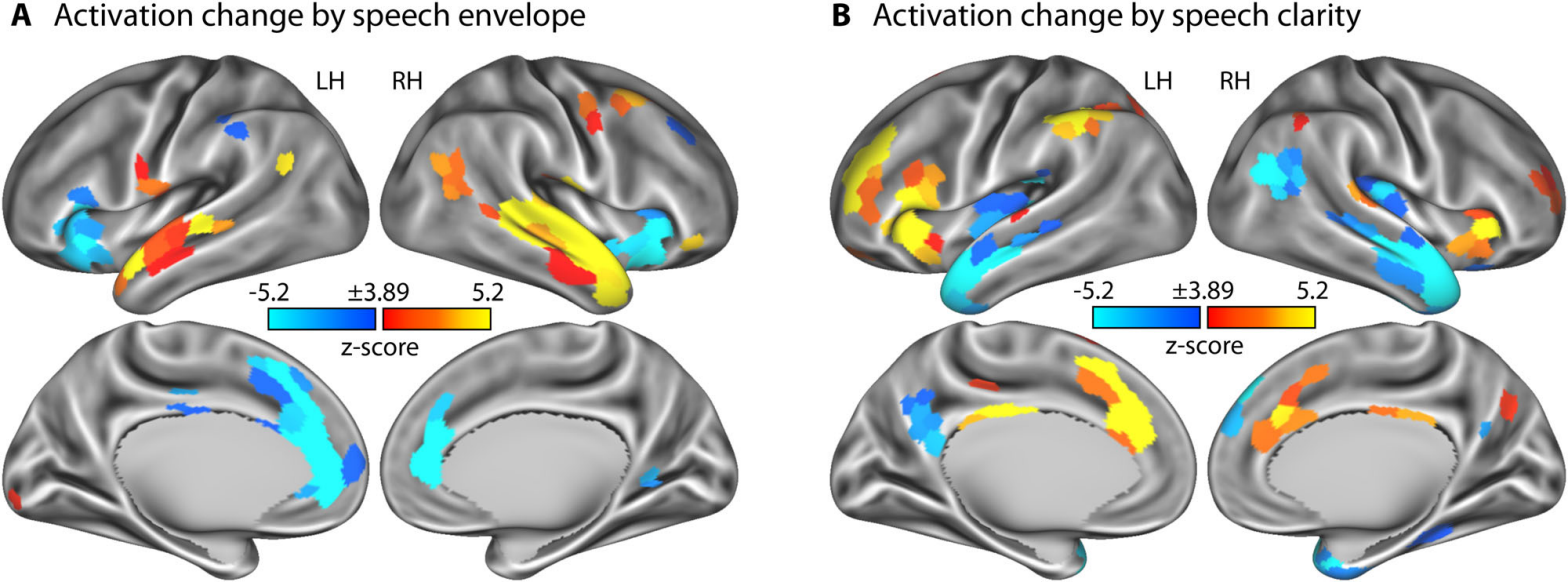
Neural activation associated with changes in the acoustic envelope of speech and speech-clarity conditions. ***A***, Statistical *z*-score map for changes in BOLD signal as a function of the speech envelope. Positive values indicate a positive correlation, whereas negative values an anticorrelation between fluctuations in the speech envelope and the fluctuations in BOLD signal. ***B***, Statistical *z*-score map for changes in BOLD signal as a function of speech-clarity conditions. Positive values indicate an increase in BOLD signal with decreasing speech clarity and associated listening effort, whereas negative values indicate an increase in BOLD signal with increasing speech clarity and associated intelligibility. *z*-score maps are thresholded at a Bonferroni-corrected 0.05 significance level, corresponding to a *z*-score of 3.89. LH, left hemisphere; RH, right hemisphere.

To first confirm that stories in our paradigm can elicit meaningful activity, we examined how the BOLD signal responds to changes in the amplitude envelope of the clear speech signal. BOLD activity fluctuations in the superior temporal cortex were positively correlated with the fluctuations in the speech envelope (i.e., activity increased as the amplitude of the speech envelope increased; Fig. 2*A*). In contrast, activity fluctuations in the left anterior insular cortex and bilaterally in the anterior cingulate cortex showed an anticorrelation with the speech-envelope fluctuations (i.e., activity increased with decreasing amplitude of the speech envelope; Fig. 2*A*). The cingulo-opercular network is thought to subserve aspects of cognitive control and has been demonstrated to be sensitive to effort during listening (Herrmann et al., 2014; Henry et al., 2015; Eckert et al., 2016; Johnsrude and Rodd, 2016). The results may thus reflect increased effort associated with the comprehension of speech in babble when the speech signal is less intense. The speech-envelope analysis thus demonstrates that our story-listening paradigm can elicit meaningful changes in BOLD activity (cf. Whitney et al., 2009; Honey et al., 2012; Rowland et al., 2018).

### BOLD activity is sensitive to the clarity of speech during story listening

Next, we investigated whether our story-listening paradigm enables us to observe intelligibility- and effort-related activations that are commonly observed in studies using sentence materials (Davis and Johnsrude, 2003; Scott et al., 2006; Wild et al., 2012a,b; Holmes and Johnsrude, 2021; Ritz et al., 2022). BOLD activity increased with decreasing speech masking (i.e., increasing intelligibility) bilaterally in auditory cortices and anterior temporal cortex and in the right posterior temporal cortex (Fig. 2*B*). In contrast, BOLD activity increased with increasing speech masking (i.e., potentially increasing effort) bilaterally in the anterior insula, the right dorsolateral prefrontal cortex, right supramarginal gyrus, and the right mid and anterior cingulate cortex (Fig. 2*B*). The regions observed in these two contrasts are consistent with those identified in previous work using sentence materials, suggesting that BOLD activity in our story-listening paradigm is sufficiently sensitive to reveal intelligibility- and effort-related activations.

### Effect of speech clarity on spatial intersubject correlation

**Figure 3.**
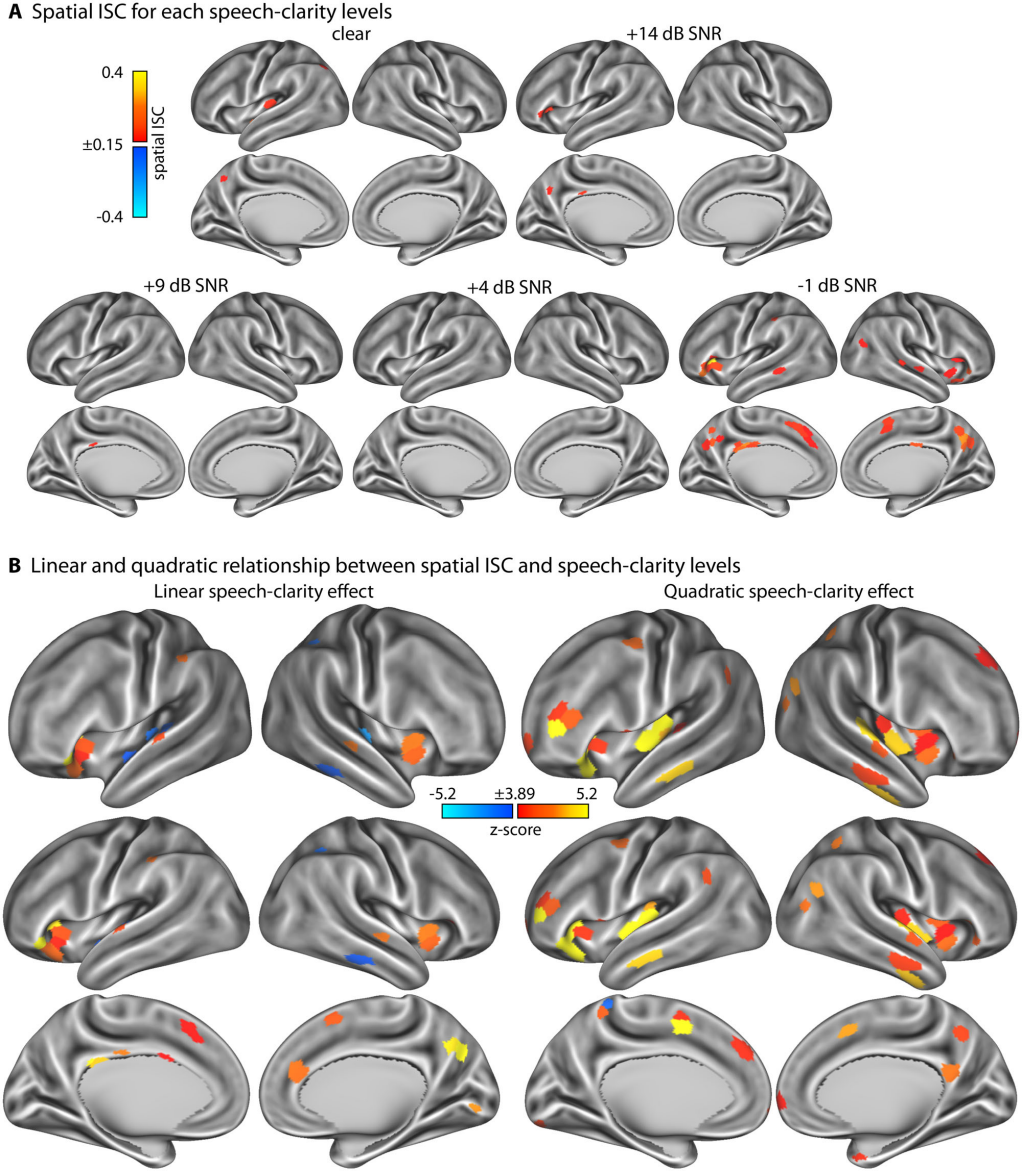
Results for spatial intersubject correlation (ISC). ***A***, Spatial ISC values for the five different speech-clarity conditions. ***B***, Statistical *z*-score maps that reflect the linear (left) and quadratic (right) change in ISC as a function of speech-clarity condition. For the linear contrast, positive values indicate an increase in spatial ISC with decreasing speech clarity, whereas negative values indicate a decrease in spatial ISC with decreasing speech clarity. For the quadratic contrast, positive values indicate an increase in spatial ISC at either both ends or one end of the speech-clarity conditions relative to the moderately masked speech conditions. *z*-score maps are thresholded at a Bonferroni-corrected 0.05 significance level, corresponding to a *z*-score of 3.89.

Spatial ISC reflects the degree to which the spatial activity patterns within a brain region are shared among listeners. Figure 3*A* shows that spatial ISC is relatively low for most speech-clarity conditions, considered on their own, with the exception of the most difficult, the −1 dB SNR, condition. For the −1 dB SNR condition, spatial ISC was strongest in the anterior insula, cingulate cortex, precuneus, and a few small regions in the temporal cortex.

The linear function fit revealed an increase in spatial ISC—that is, spatial patterns of BOLD activity were more synchronized across listeners—in the precuneus, cingulate cortex, and anterior insula as speech clarity decreased (Fig. 3*B*, left). The latter three regions are associated with listening effort (Wild et al., 2012b; Eckert et al., 2016; Johnsrude and Rodd, 2016; Peelle, 2018; Ritz et al., 2022). Spatial ISC decreased with decreasing speech clarity in a few auditory regions on the superior temporal plane and the temporal cortex (Fig. 3*B*, left).

The analysis of quadratic spatial ISC trends also revealed a positive trend in the anterior insula, capturing the fact that spatial ISC increased in the anterior insula mainly in the most difficult condition (−1 dB SNR; Fig. 3*B*, right). Spatial ISC also showed a quadratic trend in auditory regions, capturing a specific increase in spatial ISC for clear speech (Fig. 3, compare *A* and *B*, right). These changes in spatial ISC were independent of overall activity differences in these regions, because the mean BOLD activity was removed prior to ISC analyses (see Materials and Methods).

### Effect of speech clarity on temporal intersubject correlation

**Figure 4.**
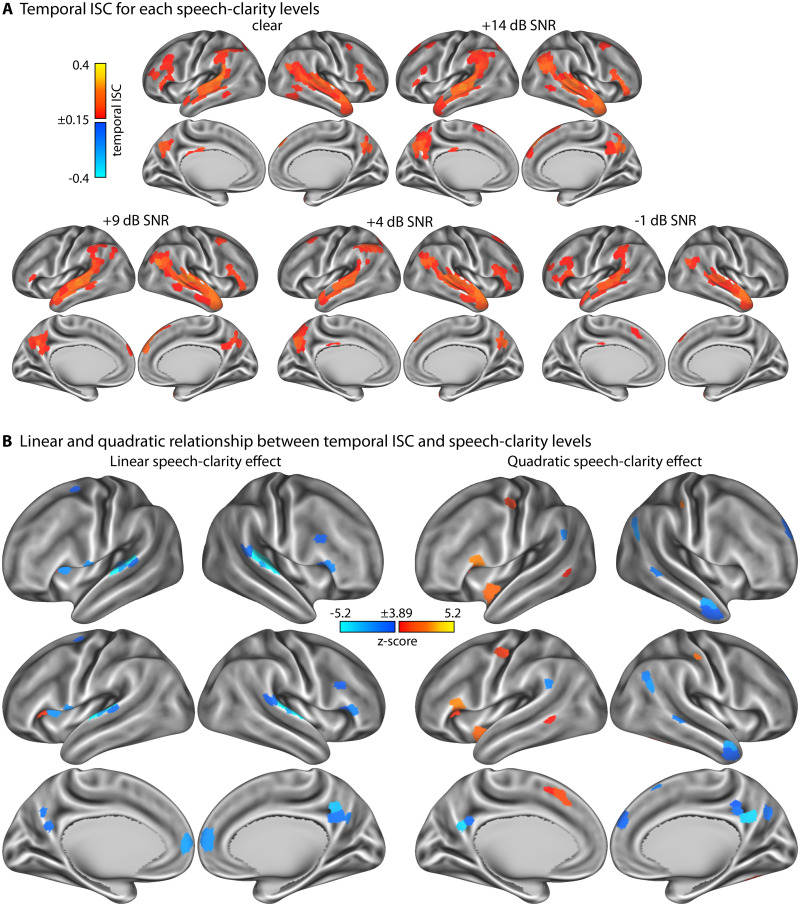
Results for temporal intersubject correlation (ISC). ***A***, Temporal ISC values for the five different speech-clarity conditions. ***B***, Statistical *z*-score maps that reflect the linear (left) and quadratic (right) change in ISC as a function of speech-clarity condition. For the linear contrast, positive values indicate an increase in temporal ISC with decreasing speech clarity, whereas negative values indicate a decrease in temporal ISC with decreasing speech clarity. For the quadratic contrast, positive values indicate an increase in temporal ISC at either both ends or one end of the speech-clarity conditions relative to the moderately masked speech conditions. *z*-score maps are thresholded at a Bonferroni-corrected 0.05 significance level, corresponding to a *z*-score of 3.89.

Temporal ISC reflects the degree to which the BOLD activity time courses evolve similarly—that is, are synchronized—across listeners. Figure 4*A* shows that activity is most strongly synchronized across listeners in bilateral superior temporal cortices and inferior frontal cortex for all speech-clarity conditions and in the precuneus in all but the most difficult condition (−1 dB SNR).

The linear function fit revealed a decrease in temporal ISC with decreasing speech clarity bilaterally in the superior temporal plane, precuneus, dorsal anterior cingulate and anterior prefrontal cortex, and the inferior frontal opercular cortex, including parts of the anterior insula (Fig. 4*B*, left). There was also a region in the left anterior insula that showed an increase in temporal ISC with decreasing speech masking (Fig. 4*B*, left), but ISC values in this region were overall very low (Fig. 4*A*).

The quadratic trend analysis revealed a positive trend for temporal ISC in relation to speech-clarity levels in the left dorsal cingulate cortex extending to the supplementary motor area, consistent with the high temporal ISC in these regions specifically for the most difficult SNR (−1 dB; Fig. 4, compare *A* and *B*, right). Additional positive quadratic trends were observed in the left inferior frontal cortex that seemed to be driven by the higher temporal ISC for the clear condition and the most difficult SNR compared with intermediate speech-clarity levels. Temporal ISC values were very low for the other few regions showing a quadratic trend (Fig. 4, compare *A* and *B*, right). The right precuneus and a few regions along the temporal cortex showed a negative quadratic trend, driven mainly by the reduced temporal ISC for the most difficult speech-clarity condition (particularly the precuneus). These changes in the temporal synchronization of neural activation across listeners are independent of overall activation differences, because the mean BOLD signal was subtracted prior to ISC analyses.

### Transient responses around event boundaries during story listening

**Figure 5.**
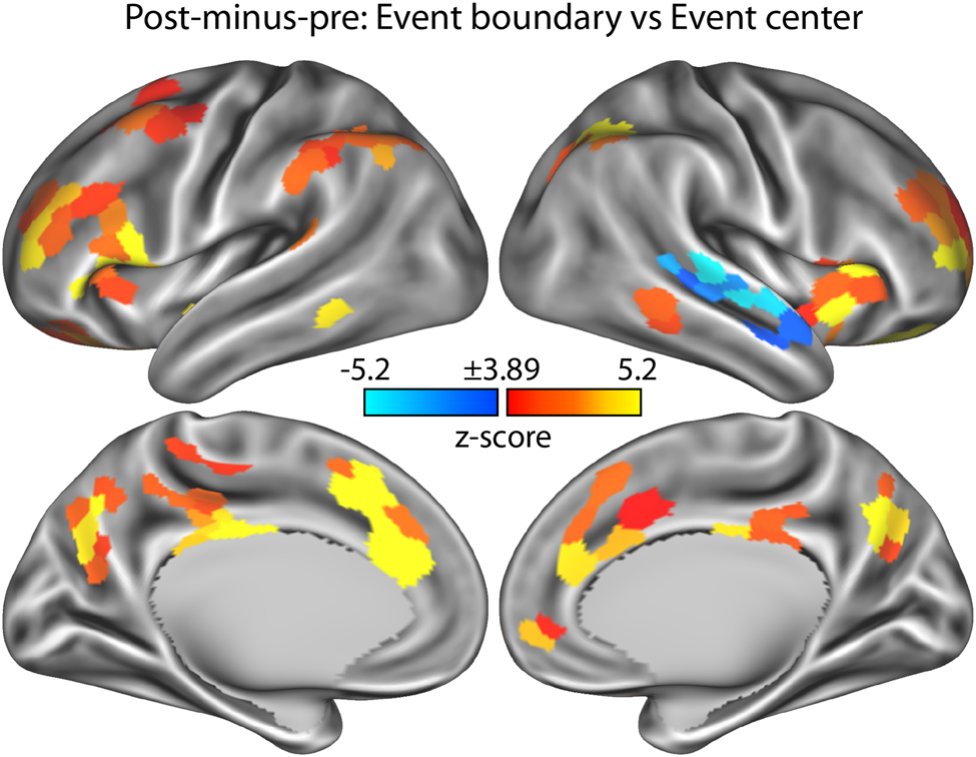
Neural activity at event boundaries. Statistical *z*-score maps show the differences in neural activation between event boundaries and event centers. The difference between the post- and the pre-time-locked windows (+1 to +7 s minus −7 to −1 s) was used as the neural activation signal for the displayed analyses. Positive values reflect a larger activation for event boundaries, whereas negative values reflect a smaller activation for event boundaries than for event centers. The *z*-score map is thresholded at a Bonferroni-corrected 0.05 significance level, corresponding to a *z*-score of 3.89. Extended Data Figure 5-1 shows similar results for a different control condition: nonboundary sentence onsets.

Neural responses to event boundaries reflect an important neural signature of event segmentation during the encoding of natural, continuous environments (Speer et al., 2007; Whitney et al., 2009; Zacks et al., 2010). We investigated changes in BOLD activity associated with event boundaries during continuous speech listening relative to BOLD activity changes associated with event centers. This analysis was conducted first across all speech-clarity conditions to establish which brain regions respond to event boundaries. Figure 5 shows the statistical *z*-score maps for differences in neural activation between event boundaries and event centers. Extended Data Figure 5-1 shows similar results for a nonboundary sentence-onset control condition.

10.1523/ENEURO.0385-25.2025.f5-1 Figure 5-1 **Neural activity at event boundaries. A and B:** Statistical z-score maps showing the differences in neural activation between event boundaries and event centers (panel A) or non-boundary sentence onsets (panel B). The difference between the post- and the pre-time-locked windows (+1 to +7 s minus -7 to -1 s) was used as the neural activation signal for the displayed analyses. Positive values reflect a larger activation for event boundaries, whereas negative values reflect a smaller activation for event boundaries than for event centers or non-boundary sentence onsets. Z-score maps are thresholded at a Bonferroni-corrected 0.05 significance level, corresponding to a z-score of 3.89. **C:** BOLD signal time courses for 7 different networks of the Schaefer brain atlas time-locked to event boundaries, event centers, or non-boundary sentence onsets. Activation time courses for the default-mode network, excluding the temporal region, and for only the temporal region of the network to better display the negative effects in temporal cortex. The shaded areas around the mean BOLD time course reflect the standard error of the mean. Solid lines close to the x-axis indicate a significant difference between event boundary vs event center or event boundary vs non-boundary sentence onset (FDR-thresholded). Download Figure 5-1, TIF file.

**Figure 6.**
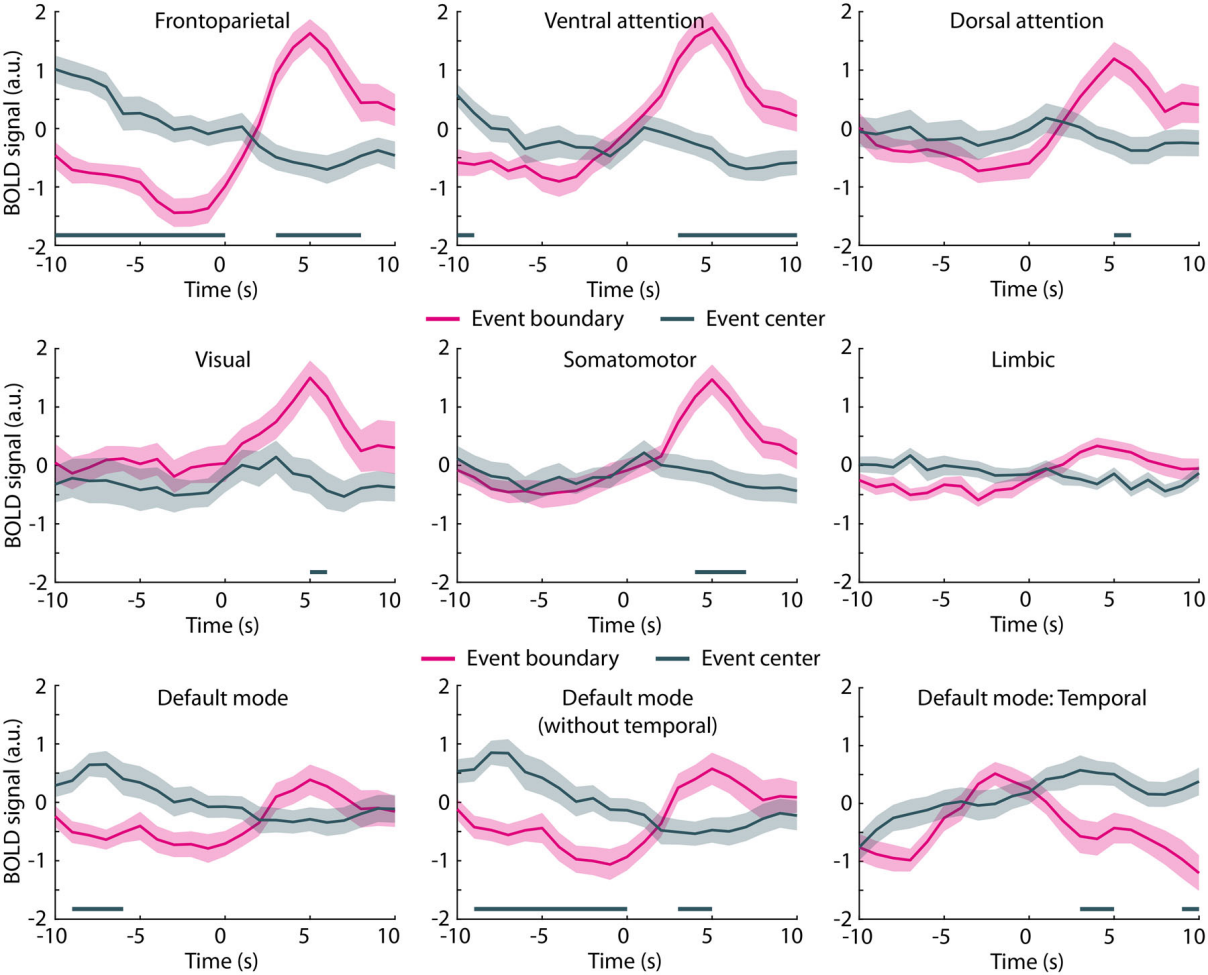
Neural activity time courses at event boundaries. BOLD signal time courses for seven different networks of the Schaefer brain atlas time-locked to event boundaries and event centers. Activation time courses for the default-mode network, excluding the temporal region, and for only the temporal region of the network to better display the negative effects in temporal cortex. The shaded areas around the mean BOLD time course reflect the standard error of the mean. Solid lines close to the *x*-axis indicate a significant difference between event boundary versus event center (FDR-thresholded).

Neural activations were larger for event boundaries compared with event centers in several brain regions that are part of the frontoparietal, ventral and dorsal attention, visual, somatomotor, and parts of the default-mode networks (Fig. 6). The frontoparietal and ventral attention networks appear to be particularly responsive to event boundaries. Activation increased and peaked at ∼5 s following an event boundary. The temporal evolution of the activation in the frontoparietal network also included an activation decrease prior to the event boundary (Fig. 6, top left). In addition to the large number of regions showing greater neural activation for event boundaries than event centers, the right temporal cortex exhibited smaller neural activation for event boundaries than event centers (Fig. 5). This effect appears to be driven by an activation peak just prior to an event boundary that was absent for event centers (Fig. 6, bottom right), resulting in the negative activation defined as the difference between the post- and the pre-time locked windows: +1 to +7 s minus −7 to −1 s.

### Change in event-boundary response associated with speech clarity

**Figure 7.**
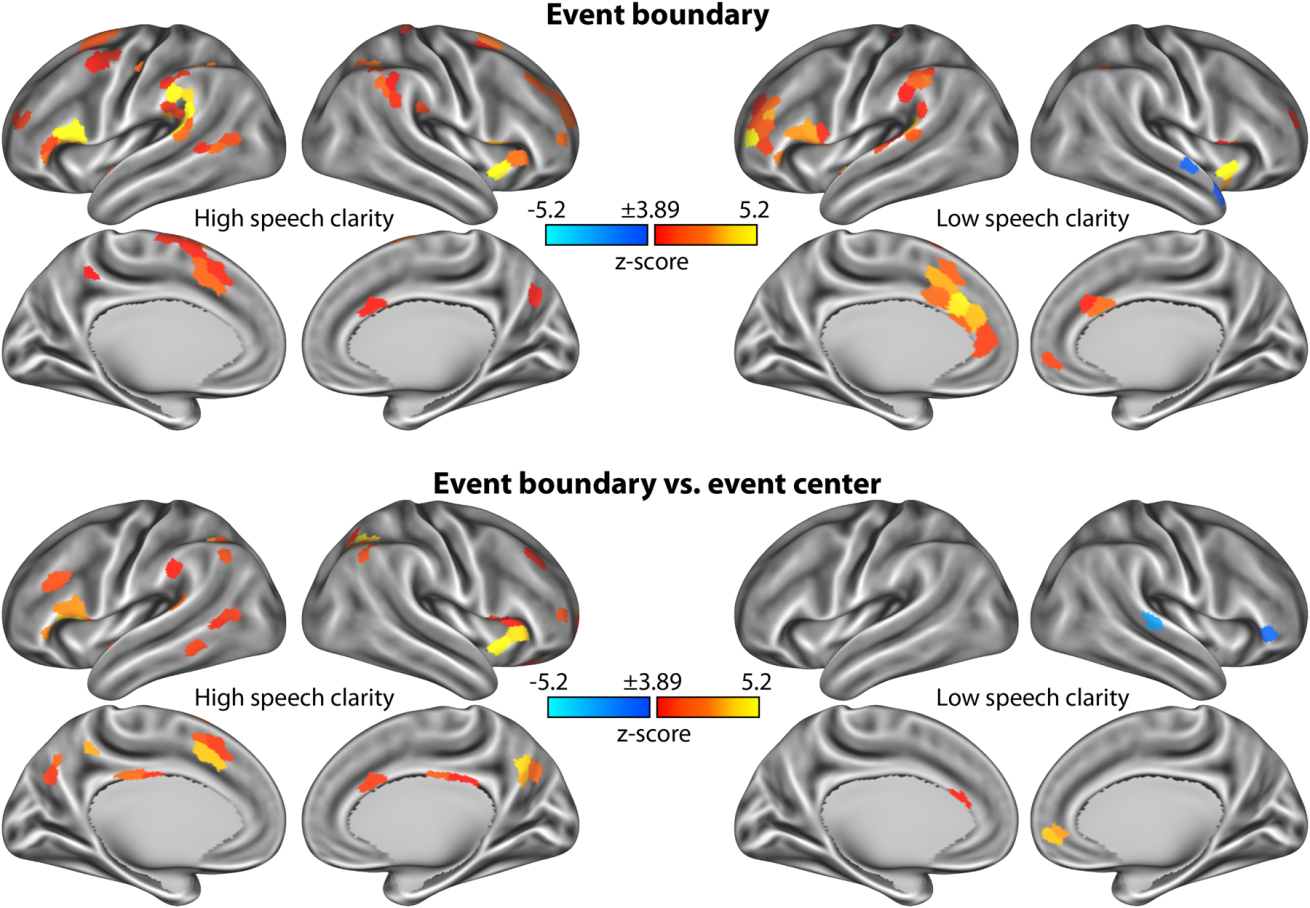
Neural activity at event boundaries, separately for low and high speech clarity. Top row, Statistical *z*-score maps showing the neural activation associated with event boundaries for high and low speech clarity. Neural activation was defined as the difference between the post- and the pre-event-boundary window (+1 to +7 s minus −7 to −1 s). *z*-score maps resulted from testing the neural activation against zero. Bottom row, Statistical *z*-score maps showing the differences in neural activation (post-minus-pre difference) between event boundaries and event centers. Positive values reflect a larger activation for event boundaries, whereas negative values reflect a smaller activation for event boundaries than for event centers. *z*-score maps are thresholded at a Bonferroni-corrected 0.05 significance level, corresponding to a *z*-score of 3.89.

To investigate how event-boundary activations are affected by speech clarity, we grouped the speech-clarity conditions according to high and low clarity. Neural activation was investigated as the difference between the BOLD signal in the post- and the pre-time-locked windows: +1 to +7 s minus −7 to −1 s. Figure 7 shows neural activation to event boundaries relative to event centers, separately for high and low speech clarity. Event boundaries elicited neural activation in the anterior insula, inferior frontal cortex, anterior cingulate cortex, dorsolateral prefrontal cortex, precuneus, posterior temporal cortex, and parietal cortex for both high and low speech clarity (Fig. 7, top row), although fewer regions were significant for low than high speech clarity (Fig. 7, bottom row).

**Figure 8.**
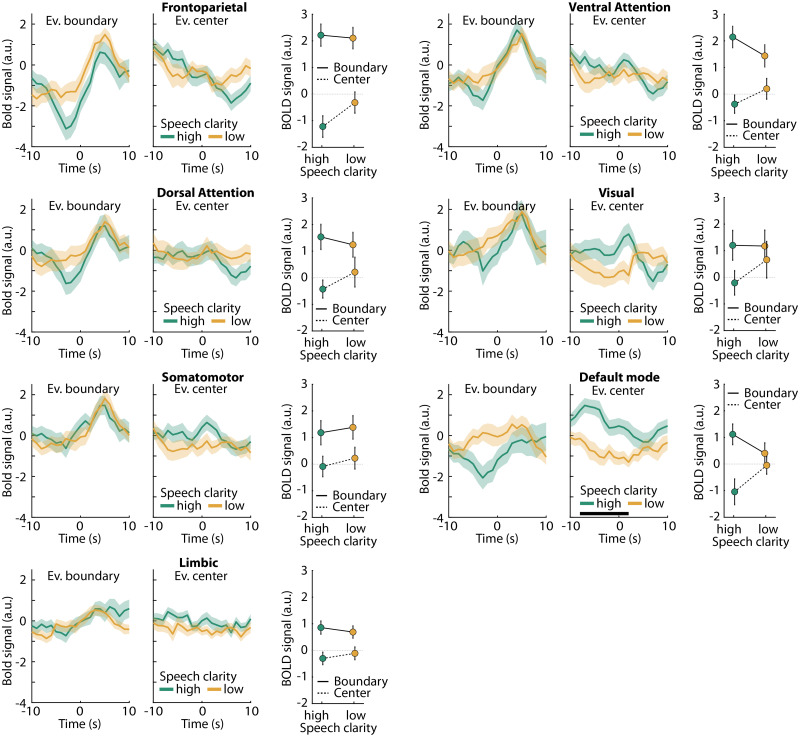
Network time courses for event-boundary responses for high versus low speech clarity. BOLD signal time courses for seven different networks of the Schaefer brain atlas. Separate time courses for high and low speech-clarity levels are shown. The shaded areas around the mean BOLD time course reflect the standard error of the mean. A black solid line close to the *x*-axis indicates a period during which high versus low speech-clarity conditions differ significantly (FDR-thresholded). Mean signals and standard error of the mean are depicted next to the time courses reflect the post-minus-pre time window difference (+1 to +7 s minus −7 to −1 s).

Directly contrasting responses for high compared with low speech clarity did not reveal a significant difference in any of the regions of the Schaefer atlas. The time courses for each brain network displayed in Figure 8 indicate that event boundaries elicit activation for both low and high speech-clarity conditions, suggesting that listeners can identify meaningful units in speech even in the presence of background masking. Activity was greater for high compared with low SNRs around event centers in the default mode network, which overlaps with the overall speech-clarity effect shown in Figure 2.

Finally, we calculated explorative repeated-measures ANOVAs to examine whether there is any indication of differences in the event-boundary response between high and low speech-clarity conditions (Fig. 8). The rmANOVAs were calculated using the post-minus-pre difference (+1 to +7 s minus −7 to −1 s) with factors Speech Clarity (high, low) and Event Type (event boundary, event center). Event boundaries elicited a larger response than event centers for all networks (for all *F*_(1,39)_ > 6, *p* < 0.02), except for the Visual Network (*p* > 0.05). However, for none of the seven networks did we observe a main effect of Speech Clarity (for all *p* > 0.35). There was no Speech Clarity by Event Type interaction (for all *p* > 0.05), except for the Default Mode network (*F*_(1,39)_ > 6, *p* < 0.02). But for the direct contrasts for the Default Mode, the event boundary response did not differ between high and low speech clarity (*t*_(39)_ = 1.101, *p* = 0.278). In fact, for none of the networks was this difference significant (for all *p* > 0.25).

## Discussion

The current fMRI study investigated how neural signatures of continuous story listening change in the presence of multitalker background babble. We show that neural activation increases in the anterior insula, anterior cingulate cortex, dorsolateral prefrontal cortex, and supramarginal gyrus when speech masking increases (Fig. 2). Temporal activation patterns in auditory brain regions became more idiosyncratic, that is, less similar, across listeners with increasing speech masking (Fig. 4). In contrast, spatial (and to some extent temporal) activation patterns were more similar across listeners in the anterior insula, anterior cingulate, precuneus, and dorsolateral prefrontal cortex when participants listened to minimally intelligible compared with highly intelligible speech, indicating shared neurocognitive processes (Fig. 3). We further observed increased activation most prominently in the frontoparietal, ventral, and dorsal attention networks at times during story listening when one meaningful event ended and another began (Figs. 5, 6). This activation to event boundaries appeared to be little affected by background masking (Figs. 7, 8). In sum, neural signatures of story listening indicate more idiosyncratic processing of the acoustic information in auditory regions but shared neural processes and intact gist processing when stories are masked by background babble.

### Neural activation associated with changes in the speech envelope and background masking

Neural activation was modulated by the fluctuations in the amplitude envelope of the speech signal, such that activity in the superior temporal cortex, putative auditory cortex near Heschl's gyrus, increased with increasing speech amplitude (Fig. 2*A*). Auditory activity correlated with changes in the speech envelope have also been reported previously in studies using fMRI (Honey et al., 2012), functional near-infrared spectroscopy (Rowland et al., 2018), and electro-/magnetoencephalography (Lalor and Foxe, 2010; Ding and Simon, 2013; Ding et al., 2014; Vanthornhout et al., 2018; Panela et al., 2024), likely reflecting the acoustic processing of the speech.

We further observed that activity increased in the anterior insula and anterior cingulate cortex with decreasing amplitude of the speech signal (i.e., showing anticorrelated fluctuations between neural activity and the speech envelope; Fig. 2*A*). Periods of lower amplitudes in the speech signal effectively mean a reduced signal-to-noise ratio given the stationary nature of the background babble. Listeners may thus need to attend more to the speech during such periods. The anterior insula and cingulate cortex, in addition to the dorsolateral prefrontal cortex, were also activated when the speech was masked by background babble (Fig. 2*B*). The cingulo-opercular network, including the insula, cingulate cortex, prefrontal cortex, and parietal cortex, is frequently observed when individuals engage in challenging tasks, including listening to degraded speech and identifying subtle changes in nonspeech sounds (Dosenbach et al., 2006, 2008; Wild et al., 2012b; Scott and McGettigan, 2013; Herrmann et al., 2014; Henry et al., 2015; Eckert et al., 2016; Johnsrude and Rodd, 2016; Peelle, 2018; Ritz et al., 2022). Increased activity in the cingulo-opercular network is thought to reflect increased executive control demands during challenging tasks (Dosenbach et al., 2006, 2008, 2025; Cole and Schneider, 2007; Crittenden et al., 2016; Gratton et al., 2018; Hausman et al., 2022). We extend this work here to story listening, demonstrating that this effort response may fluctuate moment to moment, reflecting the dynamic demands of the listening situation.

We further found increased activation in the anterior temporal cortex, precuneus, and posterior temporal cortex as speech-clarity and hence intelligibility increased (Fig. 2*B*), which is consistent with previous sentence-listening paradigms (Narain et al., 2003; Scott et al., 2006; Obleser et al., 2007, 2008; Wild et al., 2012a; Evans et al., 2016; Holmes and Johnsrude, 2021) and with the idea that these areas subserve suggested language-specific processes (Fedorenko and Thompson-Schill, 2014; Fedorenko et al., 2024).

The current data thus show that a listening paradigm with ∼10 min stories can reveal activations that are comparable with more traditional sentence-listening paradigms, with the advantage that stories are more enjoyable for listeners and enable investigating questions about time-varying and more naturalistic speech processes as described below (see Evans et al., 2016 for ∼20 s narratives).

### Changes in shared neural activity patterns as speech masking increases

The current study investigated the extent to which individuals share neural activity patterns while listening to spoken stories in the presence of background babble. We focused on the similarity of the temporal evolution of neural activity and the spatial similarity of neural activity profiles across voxels within a brain region (temporal and spatial intersubject correlation, respectively; Hasson et al., 2010; Nummenmaa et al., 2018; Nastase et al., 2019).

Temporal ISC was greatest along the superior temporal cortex, inferior frontal cortex, and precuneus for all speech-clarity conditions, consistent with previous work on story listening under clear conditions (Honey et al., 2012; Regev et al., 2019). Temporal ISC decreased with increasing speech masking in auditory regions in the superior temporal plane (Fig. 4). Reduced temporal ISC in the superior temporal plane may reflect more idiosyncratic processing and temporal tracking of the acoustic speech properties (Honey et al., 2012; Rowland et al., 2018). Temporal ISC also decreased in the posterior medial cortex (precuneus) and medial prefrontal cortex as masking level increased. Both of these regions have been linked to engagement and shared experiences with movies or spoken narratives (Lerner et al., 2011; Schmälzle et al., 2015; Simony et al., 2016; Nguyen et al., 2019; Song et al., 2021; Stawarczyk et al., 2021), potentially suggesting the experiential tracking of the narrative over time becomes more idiosyncratic when speech is highly masked, perhaps reflecting individual differences in compensation for degradation. Overall, however, temporal ISC was distributed widely over the cortex for all speech-clarity conditions, and this is consistent with attentive story listening (Regev et al., 2019) and story comprehension (Honey et al., 2012).

Although the same twelve-talker babble masker was presented across participants within each group, it is unlikely that shared masker characteristics drove the observed intersubject correlations. The babble masker is a relatively stationary acoustic background that does not contain discernible speech elements and thus does not support perceptual tracking of individual voices, words, or even syllables (Bilger, 1984; Bilger et al., 1984; Wilson, 2003; Wilson et al., 2012). Tracking such a masker would require suppressing the target speech, which would have resulted in poor story comprehension; however, participants with low comprehension scores were excluded. Moreover, masker-related shared low-level acoustic responses in auditory cortex cannot account for the observed temporal ISC patterns. Temporal ISC in auditory regions of the superior temporal plane decreased, rather than increased, with greater speech masking, that is, when the masker dominated the acoustic scene. If participants had been synchronously tracking the masker, temporal ISC should have been higher under these low-SNR conditions. We observed the opposite pattern, which supports the interpretation that ISC primarily reflects shared processing of the speech signal and narrative content, rather than correlated responses to the common background noise.

Spatial ISC was relatively low for all speech-clarity conditions considered individually, except for the most difficult SNR (Fig. 3) and in auditory regions for clear speech. That only clear speech, but none of the babble-masked speech conditions, yielded reliable spatial ISC in auditory regions is consistent with the increased idiosyncratic activity pattern in auditory regions observed also for temporal ISC. Interestingly, spatial (and to some extent temporal) ISC increased in the precuneus, anterior insula, and anterior cingulate with increasing speech masking, particularly for the minimally intelligible condition (−1 dB SNR). The latter two brain regions comprise the “cingulo-opercular network” which has frequently been implicated in executive, cognitive control and with listening effort during speech comprehension (Fig. 2*B*; Wild et al., 2012b; Herrmann et al., 2014; Henry et al., 2015; Eckert et al., 2016; Johnsrude and Rodd, 2016; Ritz et al., 2022). The increase in spatial ISC in the cingulo-opercular regions may indicate a similar mode of processing challenging speech. ISC in the posterior medial cortex (precuneus) has been linked to engagement and shared experiences (Schmälzle et al., 2015; Simony et al., 2016; Nguyen et al., 2019; Song et al., 2021; Stawarczyk et al., 2021). The increase in spatial ISC in the precuneus is surprising given the concurrent decrease in temporal ISC in this region, although the two appear to spatially dissociate somewhat along the anterior (temporal ISC) to posterior (spatial ISC) axis. We speculate that the decrease in temporal ISC reflects more idiosyncratic narrative tracking over time, whereas the increase in spatial ISC may be due to similar tonic attentional engagement across participants, as the difficulty to comprehend speech increased.

### Neural responses to event boundaries are little affected by speech masking

We observed increased neural activation in a large number of parcellated brain regions, covering frontoparietal, ventral attention, and dorsal attention networks, for story event boundaries relative to event centers and nonboundary sentence onsets (Fig. 5). This is consistent with previous research demonstrating event-boundary-related increases in neural activation during movie watching, narrative reading, and story listening in quiet (Speer et al., 2007; Whitney et al., 2009; Zacks et al., 2010; Ben-Yakov and Henson, 2018; Reagh et al., 2020; Stawarczyk et al., 2021). The activation increase at event boundaries is thought to index the updating of mental representations at an event boundary (Speer et al., 2007; Whitney et al., 2009; Zacks et al., 2010) and the magnitude of the event-boundary response has been linked to event memory (Ben-Yakov and Dudai, 2011). Moreover, accurate event segmentation requires a listener to comprehend the gist—and thematic evolution—of the ongoing story. The event-boundary response could thus potentially be used as an index of gist comprehension during listening.

The temporal evolution of the activations around event boundaries were characterized by relatively lower activity prior to event boundaries (compared with the event centers and nonboundary sentence onsets), particularly in the frontoparietal network, and peak activity ∼5 s after event-boundary onsets. The preboundary activity decrease and the hemodynamic response delay of ∼4–5 s (Buckner, 1998; Lindquist et al., 2009; Taylor et al., 2018) suggest that the response may reflect the anticipation of an event boundary rather than a reaction to the event boundary. Little or no information about a thematic change in the narrative that marks a new event was available to the listeners by the time the neural response was elicited. This may suggest that participants use prior knowledge about stories and information from event endings to predict upcoming event boundaries during story listening, which, in turn, drive the response. Preliminary work from our lab indeed suggests that participants identify event boundaries when they recognize a meaningful unit ending rather than a new meaningful unit beginning during story listening (Lamekina et al., in prep). That listeners may anticipate an event ending is also in line with recent conceptual advances suggesting that event segmentation—and subsequent memory updating—can be driven by prediction uncertainty and not only by prediction error elicited by a thematic narrative shift (Franklin et al., 2020; Nguyen et al., 2024; Smith et al., 2025).

Event boundaries are often identified through human raters (Zacks et al., 2006; Kurby and Zacks, 2011; Sargent et al., 2013; Kurby et al., 2014; Lee and Chen, 2022; Pitts et al., 2022; Sasmita and Swallow, 2022), but recent research shows that modern large language models (LLMs) can identify event boundaries in stories similarly well (Michelmann et al., 2025; Panela et al., 2025). Our observation of larger activation to LLM-identified event boundaries than to event centers (Figs. 5, 6) provides additional evidence that event boundaries identified through large language models are meaningful to human listeners as indexed through their brain responses.

Speech perception is often assessed as speech intelligibility, that is, as the verbatim word report of sentences, where speech intelligibility declines with decreasing speech clarity due to speech masking (Samar and Metz, 1988; Wilson, 2003; Wilson et al., 2012; Fontan et al., 2015; Irsik et al., 2022b; Pandey and Herrmann, 2025). Yet, our mental representations of speech are typically not verbatim but reflect the gist of larger segments of speech (Gomulicki, 1956; Mehler, 1963; Mehler and Miller, 1964; Fillenbaum, 1966; Sachs, 1967). In the current study, we observed no difference when directly comparing the event-boundary response between high and low speech clarity (Fig. 8), although listening to the stories under low speech clarity led to fewer regions that significantly responded to event boundaries compared with listening under high speech clarity (Fig. 7). Many brain regions in frontal cortex and in midline structures exhibited an event-boundary response for both high and low speech clarity (Fig. 7). The results of the current study potential suggest that background masking does not overly affect the comprehension of the gist of the story and thus event-boundary related processes. We have previously shown in behavioral and neural work that individuals remain engaged and find stories of the kind played here absorbing even when speech is presented in background noise and intelligibility is reduced (Herrmann and Johnsrude, 2020b; Irsik et al., 2022b). The activation increase for event boundaries was present even for speech at low speech clarity levels (that lead to >20% reduced speech intelligibility; Irsik et al., 2022b), suggesting that individuals can identify meaningful units under such conditions, which perhaps helps them to stay engaged in listening when speech is interesting to them.

### Conclusion

The current fMRI study reveals a diverse set of changes in brain activation patterns while individuals listened to naturalistic, spoken stories masked by different degrees of multitalker background babble noise. Spatial and temporal activation patterns in auditory regions became more idiosyncratic to individual listeners as background babble increased. However, spatial activation patterns were more similar across participants in brain regions associated with executive control and effort, indicating that shared processes are recruited during challenging listening. We further show that neural activation at the boundaries between meaningful events in a story increased in a large number of brain regions, most prominently in the frontoparietal and dorsal/ventral attention networks. There was little evidence of a decline in the event-boundary response when speech was moderately masked by background babble. Overall, the current results suggest that although the processing of acoustic information is more idiosyncratic under background babble, processing of a story's meaning is little affected by background masking (as indicated by event-boundary response), even when listening is effortful. The data may indicate that people stay engaged during story listening and that several cognitive processes associated with speech processing—potentially including processes reflecting engagement and enjoyment related to anticipation, suspense, and empathy—remain intact even under background noise.

## Data Availability

Consent for public data sharing was not obtained from participants when study data were recorded, and the data thus cannot be made publicly available. Story materials and other information can be found on the OSF repository (https://osf.io/2z7vr/).

## Synthesis

Reviewing Editor: Anne Keitel, University of Dundee Decisions are customarily a result of the Reviewing Editor and the peer reviewers coming together and discussing their recommendations until a consensus is reached. When revisions are invited, a fact-based synthesis statement explaining their decision and outlining what is needed to prepare a revision will be listed below. The following reviewer(s) agreed to reveal their identity: Samuel Evans, Ryan O'Leary. Note: If this manuscript was transferred from JNeurosci and a decision was made to accept the manuscript without peer review, a synthesis may not be available.

The manuscript has been reviewed by two expert reviewers (not the original ones) and the editor. The comments were enthusiastic, and the reviewers acknowledged the extensive revisions made after receiving comments in the previous round of reviews. One reviewer had some final minor suggestions, which were supported by the other reviewer and editor during the discussion. The decision reached was therefore "Revise - Editor Review Only".

You find the reviewer comments below. Please respond to the comments in a point-by-point manner.

*****

Reviewer #1

Advances the Field (Required)

This a well written paper that uses advanced MRI methodology to address a novel research question. Much of what we know about the neural basis of degraded speech comprehension comes from studies of sentence processing that lack ecological validity. This study investigates speech intelligibility in a more naturalistic way. The study includes aspects of "replication" in demonstrating that similar neural systems are engaged during naturalistic listening - which is reassuring. Crucially, it advances the field by leveraging advanced methods, e.g. intersubject correlation, to describe the neural effects associated with recognising event boundaries within a longer narrative, which would not be possible with spoken sentences or passages. They find that neural activity associated with this effect is largely unaffected by noise and so seems consistent with understanding of the gist/broader story arc. This provides insight on the neural basis of a qualitatively different "type" of listening behaviour. Equally the finding that neural activity becomes more idiosyncratic across participants in perceptual regions and more similar across participants in areas associated with cognitive control with increasing noise is an interesting finding. I believe that this work will act as a springboard for further research on the neural basis of naturalistic listening behaviours.

Extended Data and Multimedia

The supplementary materials detailing further analyses are useful.

Comments to the Authors (Required)

I have read the manuscript, supplementary materials and the previous reviews from another journal. My assessment is that this is a well written manuscript. It is methodologically sound and will advance the field. By using intersubject correlation the authors ask interesting new questions about the neural basis of listening in noise during "naturalistic listening". Using this approach, the authors are able to investigate listening behaviours that take place over a longer timescale (e.g. event boundaries) and to track how noise affects between-subject variability in neural responses in the spatial and temporal domain. The findings are novel and compelling in showing: (1) evidence of neural sensitivity to event boundaries that are little affected by competing noise and (2) differences in more idiosyncratic vs. shared neural activity across participants associated with listening in noise in perceptual vs. cognitive control regions.

I note that the authors have revised the manuscript addressing all of the comments from previous reviewers. The previous reviews were thorough and appropriate, and the authors have done a good in addressing the previous critiques. The previous reviewers suggested that the significance of the work/theoretical contribution of the study was not clear. In this revised version, the unique contribution of the work is now much more clearly stated and, in my view, it does represent a genuine advance. The key finding is the neural localisation of a qualitatively different "type" of listening behaviour which takes place over a longer timescale, and which was not observable in previous work on degraded speech perception. The new analyses on the boundary effects in the revised version of the manuscript bolster the previously reported findings showing that noise has minimal effect on neural activity associated with processing these boundaries. The only additional analysis that was not conducted by the authors was the suggested functional connectivity analysis. However, I agree with the authors that these additional analyses are not within the scope of this paper and that the paper is sufficiently complex without adding them.

My assessment is that the methodology and analyses are appropriate, and that the results are interpreted with sufficient qualification. Given the significant amount of work completed by the authors to improve the manuscript in the light of the previous reviews, I only have a few minor suggestions for improvements. These are to aid readability and to ease understanding for readers:

Minor issues

1. The temporal ISC analysis is clearly motivated in the introduction. However, the spatial ISC analysis could be more explicitly motivated.

2. It may be useful to expand the visualisation in Figure 1A to make the figure more useful. For example, by illustrating the envelope that is being tracked in the analyses and to describe visually to the reader the randomisation of speech in noise levels and assignment of participants to these randomisations.

3. It would be useful for unfamiliar readers to include an image of the full Schaefer parcellation to complement the network renderings in Figure 1B.

*****

Reviewer #2

Advances the Field (Required)

This paper represents a meaningful step toward bridging our understanding of speech-in-noise processing and naturalistic communication. As I mentioned in my comments to the authors, typical imaging work on more naturalistic stimuli (stories, movies, communications) is done without the consideration of the quality of the speech signal. In contrast, speech-quality work that focuses on single sentences ignores the complexity of natural communication. This is illustrated by the finding that event boundary responses are not substantially influenced by the clarity of the signal. A typical speech-in-noise researcher might assume that event boundaries would be impaired due to increased effort. However, the current work hints toward a resilience of the human discourse processing system in naturalistic listening, potentially due to the rich multi-sentence context provided by such materials.

Extended Data and Multimedia

The authors provided extended data at the request of the prior reviewers. These materials are integral to the reproducibility of the methods.

Comments to the Authors (Required)

The present manuscript reports an investigation into speech processing using sustained continuous stories as stimuli, which are more representative of naturalistic speech processing than typical single-sentence materials. The authors manipulated speech clarity using a quasi-stationary low-informational masker (12-talker babble) at 5 SNR conditions, representing a range of ~55-95% intelligibility as determined by a prior study. Analysis of change in activation corresponding to GPT-4 defined event boundaries revealed changes in several regions, with increases in activation especially prominent in frontoparietal and ventral attention networks. Interestingly, the event boundary response was not substantially impacted by the clarity of the speech signal. The authors also examined both spatial and temporal inter-subject correlations. These analyses revealed that activation in auditory regions becomes more dissimilar across listeners as masking increases, whereas activation in the cinguloopercular network (commonly thought of as an effort network) becomes more similar across listeners as a function of speech clarity. Overall, I found the approach very interesting, and I believe that the manuscript offers contributions to the literature that are both clear and novel.

To my knowledge, this is the first paper that has attempted to integrate fMRI work on processing naturalistic stories and event boundaries with the literature on speech in noise processing. Each of these domains has carried assumptions that limit their ecological validity. That is, work on naturalistic story processing typically examines stories presented in quiet, even though everyday listening is frequently challenged by background noise. On the other hand, studies of speech-clarity/ speech-in-noise processing are almost always conducted at the single-sentence level, which is not reflective of typical communication. This paper is an important step toward bridging these two domains. For example, the finding that the neural response to event boundaries is little impacted by increases in background noise is an interesting demonstration of the robustness of human discourse/gist processing, potentially indicating a resilience that would not be seen in a sentence-level experiment.

I received a revised version of this manuscript alongside comments from two previous reviewer reports. The main comments were requests for additional clarification on the theoretical motivation/novelty, requests for details on the methodology that included a request to make the story materials openly available to researchers, and some minimal suggestions for additional analyses. The authors have made substantial changes to the manuscript to accommodate these concerns. In my eyes, these concerns have been addressed, and I recommend the current manuscript for acceptance.
