# Supplementary figures and images for: Neural Signatures of Engagement and Event Segmentation during Story Listening in Background Noise
Source: eNeuro. 2026 Jan 9;13(1):ENEURO.0385-25.2025. doi: 10.1523/ENEURO.0385-25.2025 (PMC12803707; doi:10.1523/ENEURO.0385-25.2025)

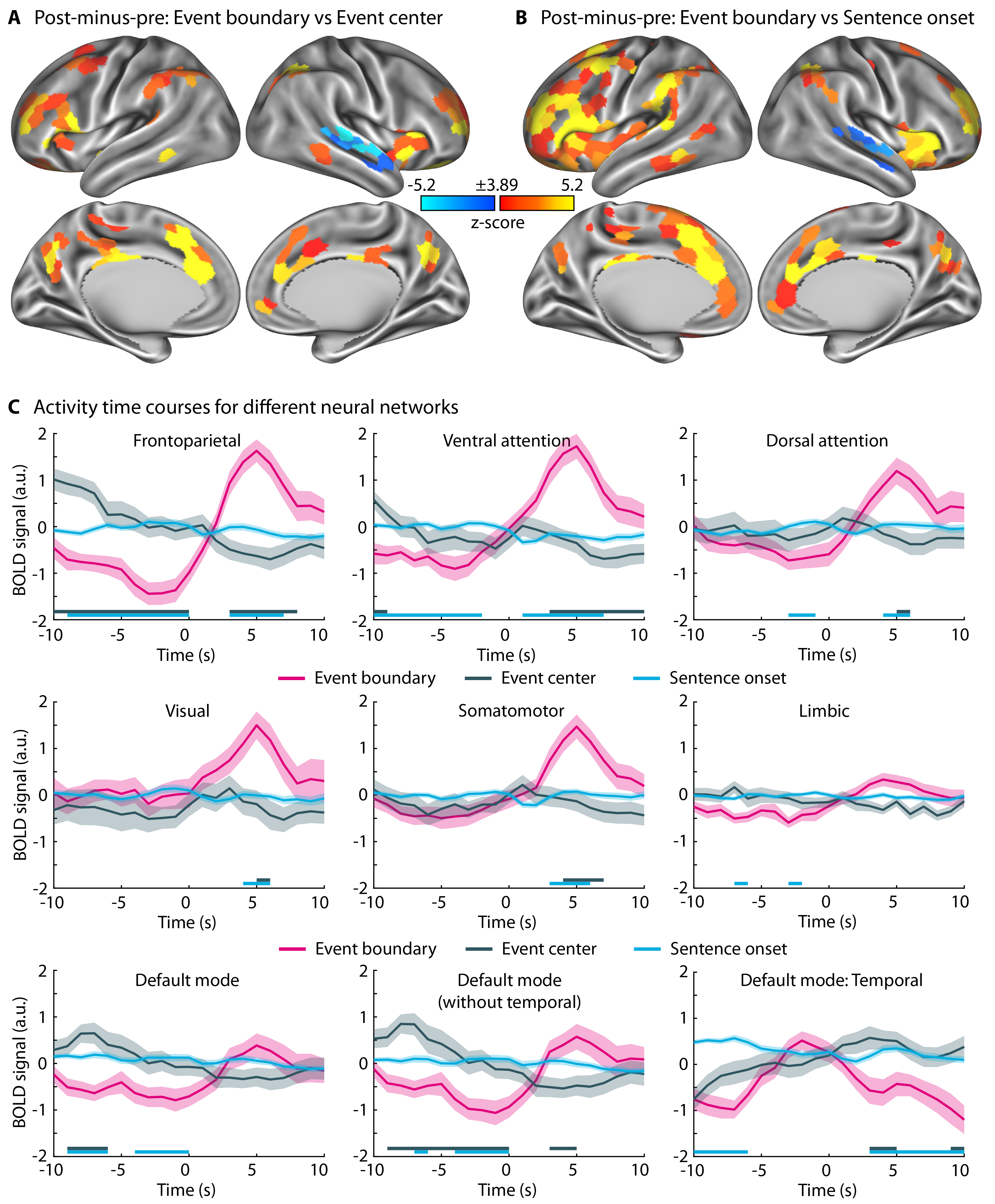

Supplement: Figure 5-1 — Neural activity at event boundaries. A and B: Statistical z-score maps showing the differences in neural activation between event boundaries and event centers (panel A) or non-boundary sentence onsets (panel B). The difference between the post- and the pre-time-locked windows (+1 to +7 s minus -7 to -1 s) was used as the neural activation signal for the displayed analyses. Positive values reflect a larger activation for event boundaries, whereas negative values reflect a smaller activation for event boundaries than for event centers or non-boundary sentence onsets. Z-score maps are thresholded at a Bonferroni-corrected 0.05 significance level, corresponding to a z-score of 3.89. C: BOLD signal time courses for 7 different networks of the Schaefer brain atlas time-locked to event boundaries, event centers, or non-boundary sentence onsets. Activation time courses for the default-mode network, excluding the temporal region, and for only the temporal region of the network to better display the negative effects in temporal cortex. The shaded areas around the mean BOLD time course reflect the standard error of the mean. Solid lines close to the x-axis indicate a significant difference between event boundary vs event center or event boundary vs non-boundary sentence onset (FDR-thresholded). Download Figure 5-1, TIF file. [file eneuro-13-ENEURO.0385-25.2025-s001.tif]
